# Exploring the attitudes of men who have sex with men on anal self-examination for early detection of primary anorectal syphilis: a qualitative study

**DOI:** 10.1186/s12879-021-06686-4

**Published:** 2021-09-20

**Authors:** Ei T. Aung, Christopher K. Fairley, Jason J. Ong, Jade E. Bilardi, Marcus Y. Chen, Eric P. F. Chow, Tiffany R. Phillips

**Affiliations:** 1 Melbourne Sexual Health Centre, Alfred Health, Melbourne, VIC Australia; 2 Central Clinical School, Faculty of Medicine, Nursing and Health Sciences, Monash University, Melbourne, VIC Australia; 3 Centre for Epidemiology and Biostatistics, Melbourne School of Population and Global Health, The University of Melbourne, Melbourne, VIC Australia; 4 Department of General Practice, Faculty of Medicine, Dentistry and Health Sciences, The University of Melbourne, Melbourne, VIC Australia

**Keywords:** Anal self-examination, Anal syphilis, Screening for anal syphilis, Syphilis in MSM

## Abstract

**Background:**

Studies show men who have sex with men (MSM) practising receptive anal sex are more likely to present with secondary syphilis, suggesting anorectal primary lesions are being missed. Regular anal self-examination might be able to detect anorectal syphilis lesions, hence potentially reducing transmission. This study aimed to explore the attitudes of MSM on performing anal self-examination to detect primary syphilis.

**Methods:**

In this qualitative study, 20 MSM over 18 years of age were purposively sampled from a sexual health clinic to participate in semi-structured interviews. Interviews were recorded, transcribed verbatim and data analysed thematically.

**Results:**

Four major themes and 12 sub-themes were generated from the study: (1) reasons for performing anal self-examination, (2) preferred educational resources for anal self-examination, (3) attitudes towards partner anal examination, and (4) acceptability of anal self-examination. Most participants had performed some form of anal self-examination in the past, and, just over half performed regularly for mostly health-related concerns. Most participants who infrequently or never performed anal self-examination were agreeable to perform regularly if it was recommended by health professionals with appropriate guidance. Participants preferred education on anal self-examination from health professionals and trusted online learning resources.

**Conclusion:**

Our study showed MSM were agreeable to anal self-examination however would like to receive education and training to gain more confidence in conducting anal self-examination as a screening tool. Further studies are required to explore the adherence and acceptability of anal self-examination for syphilis prior to studies examining efficacy. The study provides foundation for any future policy aiming at utilising anal self-examination as a screening tool for syphilis among MSM.

**Supplementary Information:**

The online version contains supplementary material available at 10.1186/s12879-021-06686-4.

## Background

The notification rate of syphilis in Australia increased between 2014 and 2018, from 9 to 20.8 notifications per 100,000 people [1]. Increases have also been seen in other high-income countries, mainly among key populations including men who have sex with men (MSM), and transgender individuals [2–4]. Over the last decades, there have been numerous public health interventions aimed at improving syphilis prevention and control, including regular testing [5, 6], improvements in contact tracing [6], and interventions to increase condom use [7], yet the notification rate of syphilis continues to increase [8]. Novel strategies for syphilis control are required [9].

One of the main strategies underpinning interventions for controlling syphilis is to reduce the duration of infectiousness. Primary syphilis classically presents as a painless chancre at the point of inoculation that results in inflammation and local swelling [10]. These primary lesions may go unnoticed if they occur at hidden sites such as the vaginal or inside the anal canal [10]. A number of case reports have been published reporting anorectal syphilis mimicking as other diseases such as anorectal cancer or Crohn’s disease [11–15]. Cornelisse et al. reported that MSM who practised receptive penile-anal sex were four times more likely to have secondary syphilis than those who practised insertive penile-anal sex, suggesting primary anorectal lesions are often missed, leading to progression to secondary syphilis [16]. In this study, 27% (77/338) of primary syphilis were at anus [16]. If men examine their anus regularly, they might be able to detect primary anorectal lesions and present for treatment before progressing to the secondary stage, thereby reducing infectiousness.

Anal self-examination, although a new concept for syphilis detection, is a practice promoted among MSM aged over 50 who are living with HIV to detect early anal cancer [17–20]. This practice is different from digital rectal examination performed by a healthcare professional. A previous qualitative study among MSM living with HIV suggested that annual anal self-examination is an acceptable approach for anal cancer screening [18]. However, there have been no studies examining the acceptability of regular anal self-examination for detecting syphilis. The aim of this study was to explore the attitudes of MSM towards anal self-examination and willingness to adopt the practice routinely for the purpose of syphilis detection.

## Methods

A Qualitative Descriptive approach was used in this study. A Qualitative Descriptive approach is a pragmatic rather than theory driven approach that is commonly used in healthcare research when researchers have questions of specific clinical interest. It aims to provide a description of participants’ experiences or events rather than a theory driven or interpretive analysis. It is a particularly useful approach to employ when little is known about a topic area or as a means of informing the development of an intervention [21, 22] in this case, the attitudes and willingness of MSM to perform anal self-examination for syphilis detection [23, 24].We adhered to the qualitative research review guidelines (RATS) (Additional file 1) in reporting the findings of this research [25].

### Ethic approval

This study was approved by the Alfred Hospital Ethics Committee, Melbourne, Australia (Project 735/19).

### Participants

The SEAS-Q (Self-Examination of Anus for Syphilis – a Qualitative study) study consisted of semi-structured face-to-face interviews conducted at the Melbourne Sexual Health Centre (MSHC), Australia between February and March 2020. MSM aged ≥ 18 years and who had practised receptive penile-anal sex in the last 12 months were eligible for the SEAS-Q study. Men who practised insertive penile-anal sex only or were not fluent in English were not eligible. To ensure maximum variation sampling, men of varying ages, countries of birth, HIV status, PrEP use, and sexual practices (see below) were recruited.

Men who practised insertive penile-anal sex were referred to as “top”, men who practiced receptive penile-anal sex were referred to as “bottom” during the interviews, and men who practiced both insertive (top) and receptive (bottom) penile-anal sex were referred as “versatile”.

### Procedure

The research team liaised with the clinicians to inform them of the eligibility criteria and the number of participants to be recruited and regularly updated the clinicians of the recruitment progress to ensure maximum sampling variation among participants with varying HIV status, PrEP use, and sexual practices. We aimed to recruit a similar number of men who were PrEP users and non-PrEP users and men not living with HIV infection and men living with HIV. We also sought to recruit men with varying sexual practices. While our eligibility criteria included men who practised either versatile anal sex or receptive anal sex, given anal syphilis is most likely to occur in men who practice mostly receptive penile-anal sex, we also wanted to gather the views of men who practised insertive anal sex on anal self-examination. To enable this, clinicians referred two men who practised mostly insertive anal sex. Eligible MSM were informed about the study and invited to participate by the clinicians during their clinic visit. If the potential participants expressed interest in the study, the clinicians, with consent, provided the details of the potential participants to the research team. A researcher (ETA) then met in person with the interested MSM to confirm eligibility and scheduled a time for a face-to-face interview, usually on the same day after the consultation with the clinicians. A patient information and consent form detailing the study, confidentiality and privacy was given and the study was explained prior to obtaining written informed consent before the interview. All interviews were conducted by ETA at MSHC and were audio recorded and transcribed verbatim. ETA is a sexual health academic clinician who undertook the research as a part of her doctoral studies. As a sexual health clinician, ETA is highly accustomed to speaking to people about sexual health, particularly with MSM, and has a sound understanding of syphilis and its pathogenesis, clinical features, and treatment. It is acknowledged that ETA’s gender (cisgender woman) and clinical background may have influenced the information participants chose to share and the interpretation of the data. However, during the study period, ETA was solely working on research projects, and not as a clinician and therefore introduced herself to participants as a researcher as opposed to a clinician to reduce any potential bias. She worked under the primary supervision of TRP (a non-clinical researcher with expertise in qualitative research) in this project, who was also involved on the research question formulation and data interpretation. All participants were required to complete a structured 11 item questionnaire about their demographic characteristics before being asked a series of open-ended questions relating to their experiences of and attitudes and willingness towards self-examination and partner examination of their anus. Participants were shown a picture describing anal self-examination and a series of pictures (Additional file 2) with different positions for anal self-examination during the interview to facilitate understanding of the procedure and to elicit participants’ preferred positions for conducting anal self-examination. An AUD $30 gift voucher was given to the study participants as compensation for their time and travel costs after the interview.

All participants’ demographic information was recorded in a password-protected file and contained only the study ID. This document along with the audio-recorded interviews were kept in a secure folder on a shared drive accessible only to members of the research team. During the interview, participants did not state their name and no identifying information was asked or shared. Participants were made aware of how their confidentiality would be maintained in the Patient Information and Consent Form, which was reviewed with them by a member of the research team prior to obtaining written informed consent.

Our study focused on anal self-examination, which is a new and unique concept, which to our knowledge has not been the topic of prior research. We had strict selection criteria which included only MSM who practiced receptive anal intercourse. After the first 3 interviews were complete, the interviews were transcribed and read to determine whether any new lines of questioning were arising from the interviews. Additional questions on partner anal examination were included in subsequent interviews. During the data collection period interviews were transcribed, read, coded and initial themes and sub-themes generated. The research team met regularly throughout this period to discuss and review the developing themes. After 20 interviews were complete, ETA and TRP met to discuss the themes and sub-themes and determined no further themes were developing from the interviews and data saturation had been met [26–30].

### Analysis

Interview data was transcribed, and transcripts were imported to NVivo for data management (QSR International Pty Ltd, version 12.6.0, 2019). Data were analysed using a Codebook Thematic Analysis (Codebook TA) approach [31]. Thematic analysis involves identifying patterns of meaning in the data in order to answer a research question. Patterns are identified by thoroughly familiarising oneself with the data, coding the data and developing and revising themes. Codebook TA uses some kind of structured coding framework for developing and reporting the analysis, with themes generally developed early on, which may be refined as analysis progresses and new themes added as they are generated inductively from the data [31]. A Codebook TA approach is often employed for pragmatic reasons and where specific information needs are required by the researchers. Data are often tangible, and the output required being a summative or descriptive analysis of results which can be actioned by clinicians or stakeholders or used to inform clinical practice [31]. Codebook TA was undertaken by ETA whereby each transcript was read and coded before a coding framework was developed. Codes were grouped into themes and sub-themes which were largely derived deductively from the interview schedule and question topics, with some additional themes generated inductively from the data. The themes and sub-themes were reviewed and further refined and compared for similarities and differences. The interview transcripts were analysed using both a deductive approach (with themes drawn from the interview schedule topics) and an inductive approach (where recurring themes were generated from the data).

A subset of transcripts was independently read and analysed by TRP. TRP and ETA then met to review the coding of the transcripts and discuss the generated themes and sub-themes, with no major differences in interpretation evident. This collaborative approach, as described by Braun et al. 2019 [32] was used to develop a more nuanced reading of the data and minimise data interpretation bias. We looked for differences in themes among three groups of participants i.e., living with HIV infection, PrEP user, not living with HIV and not taking PrEP, and between those who had ever performed anal self-examination and those who had never performed anal self-examination.

## Results

Twenty-two men were referred to the research team; of these two declined to participate due to time constraints and twenty men participated in the study. Interviews were an average length of 24 min (range 15–34 min).

The age of the participants ranged from 21 to 53 years, with a median age of 31 (interquartile range [IQR] 27–39) years. The demographics of the participants are shown in Table 1.

**Table 1:** Demographics of 20 participants

	N = 20	Percentage (%)
Sexual practice		
Practised receptive anal sex (bottom) only*	2	10
Practised versatile anal sex^#^	18	90
HIV status and PrEP use		
Living with HIV and on treatment	6	30
Not living with HIV and taking PrEP	7	35
Not living with HIV and not taking PrEP	7	35
Country of birth		
Australia	10	50
Overseas	10	50
Language at home		
English	15	75
Others (Chinese, Lebanese, Portuguese, French, Spanish)	5	25
Self-reported previous syphilis diagnosis	10	50

*Bottom or receptive anal sex: receptive partner in an anal sexual intercourse

^#^Versatile anal sex: receptive and insertive partner in an anal sexual intercourse

### Anal self-examination

Anal self-examination was defined as regular based on participants’ perception of whether they were performing anal self-examination regularly or infrequently. Three groups of men were generated in this study: those who were already performing regular anal self-examination, those who infrequently or occasionally performed anal self-examination and those who had never performed anal self-examination. Men who were performing anal self-examination occasionally were not familiar with the term “anal self-examination”, however, after explaining the procedure using pictures of anal self-examination, most participants reported having performed similar examinations previously. Among the three men who had never performed anal self-examination, none knew about performing anal self-examination for health reasons before the interview. These three men, who were not living with HIV, had no previously known abnormalities in their anus and all of them practised versatile anal sex position.

Overall, most participants had performed some form of anal self-examination at least once previously (17 men); among these men, half were regularly performing anal self-examination (11 men) and about a third occasionally performed anal self-examination (six men).

As part of the interview, men were shown four potential positions that they would be likely to adopt during anal self-examination (Additional file 2). Most participants preferred to lie on their side for anal self-examination, while some reported they preferred squatting and standing positions. A couple of men reported performing anal self-examinations while lying on their back with legs raised. The participants reported performing anal self-examination mostly in the shower and toilet for standing and squatting positions, followed by in the bedroom for lying positions.

### Themes

Four major themes and 12 sub-themes were generated from the study: (1) Reasons for performing anal self-examination, (2) Preferred educational resources for anal self-examination, (3) Attitudes towards partner anal examination, and (4) Acceptability of anal self-examination (Table 2).

1. **Reasons for performing anal self-examination**

**Table 2:** Major themes and subthemes

Major themes	Subthemes	Number of participants (N = 20)
1. Reasons for performing anal self-examination	Health concerns (anal cancer screening, screening for anal pathologies and STI, medical treatment, being anal health conscious)	17
	Hygiene/cleaning	3
	Sexual stimulation/masturbation	6
2. Preferred educational resources for anal self-examination	Self-learning through online resources or self-practice	10
	Learning through healthcare professionals and trusted organizations (Thorne Harbour Health, Melbourne Sexual Health Centre)	10
3. Attitudes towards partner anal examination	Willing to have partner anal examination if in a trusted or stable relationship	10
	Not comfortable with partner anal examination	10
4. Acceptability of anal self-examination	Acceptable to perform regular anal self- examination if recommended by a health professional	10
	Open to perform anal self-examination when proven effective	4
	Already performing regular anal self-examination for health concerns	6
	Preference for a health professional performing anal examination rather than self-examination	7
	Challenges of anal self-examination as a means for syphilis detection	6

Some subthemes might not add up to total 20 as multiple subthemes can be elicited from an individual participant

**Fig. 1:**
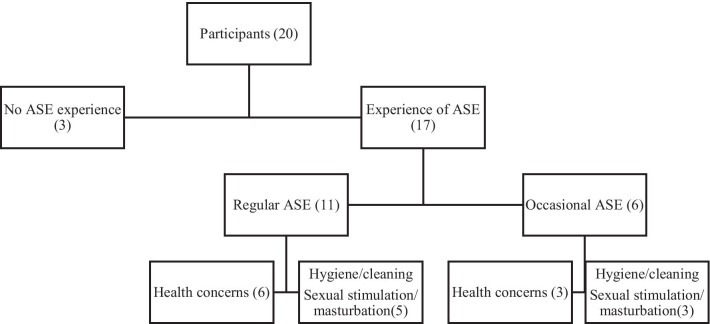
Reasons for performing anal self-examination among 20 participants. *ASE* anal self-examination

Reasons for performing anal self-examination varied, but generally involved concerns about symptoms related to sexually transmitted infections (STI). The reasons that men who had ever performed anal self-examination are shown in Fig. 1.

#### Health concerns

Most men who regularly or occasionally performed anal self-examination did so for health reasons. Some men regularly checked their anus for signs of STIs, while some were prompted by previous experiences of non-STI anal problems such as haemorrhoids or fissures or specific STI events such as genital herpes, syphilis, or genital warts infections. When asked about their main concerns, most men worried about STIs and some men, mostly those who were living with HIV, were concerned about anal cancer. A few men expressed feeling distressed on finding an abnormality, while other men reported they would seek medical advice instead of worrying.

Most men who regularly performed anal self-examination for health concerns felt performing anal self-examination regularly was a way to maintain their anal health.

*“I would say like once every month, I try to do a rectal examination myself, just to check that there are no warts, that there’s not anything that I have to worry about…”*

—Participant 20, not living with HIV and not taking PrEP

Among the occasional or infrequent anal self-examination users, most had performed anal self-examination as a once off or within a limited timeframe in response to a specific health concern. While the health concern that prompted a period of anal self-examination varied among participants, men in this category described examining their anus in response to symptoms suggestive of an STI or treatment required for anal warts. The time frame of practising anal self-examination varied; men who checked their anus for STI symptoms described performing an exam as a once-off as a way to investigate an unpleasant pain or burning in their anus, while those who were taking anal warts treatment described examining their anus while inserting the medication and checking for the continued existence of warts in the anus for a period of time during treatment.

#### Sexual stimulation/masturbation

Around a third of men reported performing anal self-examination for sexual pleasure. These men examined their anus incidentally while pleasuring themselves. The frequency with which these men inserted their finger into their anus for pleasure varied, but all described being able to tell if they felt an abnormality.

*“I have done that for sexual pleasure, but not for medical purposes…I’m certain that if I—because I am sexually active, I’m sure I would have noticed if there was something that didn’t feel normal.’*

—Participant 4, not living with HIV and taking PrEP

#### Hygiene/cleaning

A few men performed anal self-examination for hygiene or cleaning purpose.

*“So, like even if I’m not having sex now I’ve still been douching to clean up and just keep checking it to make sure that it’s not bigger or smaller or there’s more around it or whatever”.*

—Participant 10, HIV negative, taking PrEP

Table 3 provides example quotes relating to the reasons for previously performing anal self-examination. Some men conducted anal self-examination for more than one reason such as checking the anus for anal pathologies and pleasure or for cleaning and pleasure.

2. **Preferred educational resources for anal self-examination**

**Table 3:** Example quotes around reasons for anal self-examination (Theme 1)

Reason for performing anal self-examination	Quote
Health concerns	
STI concerns	*“I will clean the area thoroughly, I might, you know put my finger up there a little bit, washing out with soap and everything normal on a daily basis, usually I don’t notice any problem. But the couple of times that I have noticed chancre before, they are easily noticeable, it’s not like they are so deep that I can’t notice casually. It is very easy for me to tell something if they shouldn’t be there.”* —Participant 1, living with HIV
STI concerns	*“I previously got what felt like a urinary tract infection, which turned out to be chlamydia and gonorrhoea in the bum. At that time, I did check it out and something didn’t feel right,…. It just felt kind of hot and inflamed sort of. I knew something was wrong, but I couldn’t find out what it was.”* —Participant 17, not living with HIV and taking PrEP
Sexual stimulation/masturbation	*“I guess when I’m just sort of playing around on my own, I would sometimes stimulate that area. So, I kind of somewhat feel like I know what would be normal and not normal feel wise.”* —Participant 11, living with HIV
Hygiene/cleaning	*“Pretty much every week, because again, when I’m trimming and grooming and stuff, you’re always quite close, so I would notice if something was abnormal.”* —Participant 4, not living with HIV and taking PrEP

Most men preferred learning from online resources, although some preferred learning from a health professional, and a few preferred learning by practising anal self-examination without any guidance from online, health professionals or friends and partners.

#### Self-learning through online resources or self-practice

Men who preferred learning anal self-examination through online resources reported accessibility as the main reason for their preference.

*“It’s the most easily accessed…. online’s probably the easiest resource cause everyone owns a phone, everyone owns a computer. You know, like it’s readily accessible.”*

—Participant 2, not living with HIV and not taking PrEP

Men who self-learned without guidance from anyone or any resources described anal self-examination as being easy to learn. These participants reported that self-exploration to teach themselves essentially required the use of “common sense” and knowing their own body helped them to distinguish between normal and abnormal findings (see Table 4 for example quotes).

**Table 4:** Example quotes around men’s preferred educational resources for learning anal self-examination (Theme 2)

Preferred educational resources for learning anal self-examination	Quote
Self-learning through online resources	*“Potentially, like, online resources, in a video format, but perhaps like a cartoon or something, so it’s not as full on to watch, and that could be like an animation of how to check that type of stuff and how to do it correctly. Yeah, that’s probably the best way I can think of.”* —Participant 5, not living with HIV and not on PrEP
Self-learning through practice	*“I think it's like picking your nose, you just sort of know how yeah. …you know where … your nose is and your knees are and your toes are, you can do it with your eyes closed. Put your finger to your spots yeah.”* —Participant 12, living with HIV
Learning through healthcare professionals and trusted organisations	*“I mean, the internet is good, but if you learn it from your GP, at least your GP can check that you’re doing it properly, and then once they’ve seen that you do it properly, then you know, you know that you’re going to do it the proper way, otherwise people can sort of watch a video but you’re never sure that they’re going to do it the way it should be done. ”* *—*Participant 19, living with HIV

#### Learning through healthcare professionals and trusted organisations

Many men indicated that they preferred having a health professional to teach them how to do anal self-examination. The majority of men who preferred a health professional teach them were men who were infrequently practising or had never performed anal self-examination and were uncertain about what to look or feel for during the examination. Their main concern was not being able to tell the difference between normal and abnormal findings and preferred a clinician teach them about anal self-examination.

*“Yeah, it doesn’t have to be too in-depth because maybe if things like abnormalities of what to feel for, although I’m pretty sure I could tell what to feel for but just somethings that I might not know. Even just a normal explanation in laymen terms just so I completely understand, rather than trying to wing it myself.”*

—Participant 14, not living with HIV and taking PrEP

Several men indicated they would like clinicians to initiate the conversation and educate them about anal self-examination but that once they knew how to perform anal self-examination properly, they would be comfortable to do it themselves. These men felt that while the internet would be helpful to learn how to do anal self-examination, a person would first need to know about anal self-examination and why it should be done before they would think to look up how to do it.

*“I think places like Pronto [peer-led community service offering HIV and sexual health screening], and places like here [Melbourne Sexual Health Centre, a sexual health clinic], just during general screenings, I think if either doctors …sort of ask the patients, you know, like, have you—or do you ever do a personal anal examination? I think you’d have so many people who sort of are a bit like me, you know, and don’t really know what the doctors mean. I guess that’s how we become educated.”*

—Participant 4, not living with HIV and taking PrEP

3. **Attitudes towards partner anal examination**

Responses were mixed in terms of men’s comfort levels with partner-assisted examination. Half of the participants (10/20, 50%) reported they were comfortable having their partners perform an anal examination on them, although they would only agree to partner examination if they were in a trusting relationship with a partner or a friend. The other half of the participants (10/20, 50%) were not comfortable having their partners examine their anus.

#### Willing to have partner anal examination if in a trusted or table relationship

Among those who were comfortable having a partner perform anal examination, trust was the main factor. Some men reported this trust came from regular partnership or friendship, whereas for others it was about a developed intimacy between sexual partners. Nevertheless, these men felt they would not ask a casual partner to examine them for fear it would be awkward. Men who reported being “open” and “liberal” regarding conversations around anal health appeared were more likely to be comfortable with having a partner administer anal examination themselves (see Table 5 for example quotes).

**Table 5:** Example quotes around attitudes towards partner anal examination (Theme 3)

Attitudes towards partner anal examination	Quote
Willing to have partner anal examination if in a trusted or table relationship	*“If we are intimate, and I trust them—but it doesn’t have to be regular [partner], but there has to be some kind of trust in that … Like, my current partner, I would ask them.”* —Participant 10, not living with HIV and taking PrEP
Not comfortable with partner anal examination	*“About medical things I would trust a doctor, about a sexual partner I would trust them for sexual things”* —Participant 12, living with HIV

#### Not comfortable with partner anal examination

Among men who were not comfortable with a partner performing anal examination, some preferred to keep their medical and sex life separate from their partners (see Table 5 for example quotes). They also placed trust in health professionals and would prefer to see a doctor about STI or sexual health-related issues. A few men felt that it was “weird” to ask someone else to do it for them when they could do the examination themselves.

*“… I could do it myself yeah it’s a bit weird to ask someone else to do it for you.”*

—Participant 16, not living with HIV and not on PrEP

4. **Acceptability of anal self-examination for syphilis detection**

Most men reported they would be willing to practice anal self-examination for syphilis detection in the future if it was recommended by a health professional, if it was proven effective or if they were already performing regular anal self-examination for health concerns. However, some men still had hesitations citing reasons such as lack of knowledge and wanting more evidence that such screening was helpful before initiating a regular practice (See Table 6 for example quotes).

**Table 6:** Example quotes around the acceptability of anal self-examination for syphilis detection (Theme 4)

Acceptability of anal self-examination for detection of syphilis	Quote
Open to perform anal self-examination when proven effective	*“If it becomes part of some evidence-based treatment guideline or prevention guideline, sure.”* —Participant 9, not living with HIV and taking PrEP
Acceptable to perform anal self-examination if recommended by a health professional	*“It's something I would be prepared to do if it was recommended by a GP or a doctor, yes”* —Participant 13, not living with HIV and not taking PrEP
Preference for a health professional performing anal examination rather than self-examination	*“I think for me, personally, it would be the fact that I wouldn’t be confident in knowing what I was looking for if I was to do it myself, and that probably would put me off doing it myself. I’d prefer someone—well, I wouldn’t really want someone else to check, like a doctor or something, but if I had to, I would, if that makes sense, because I know they know what they’re looking for.”* —Participant 5, not living with HIV and not taking PrEP
Already performing regular anal self-examination for health reasons	*“I think so [it would be acceptable], because anything that is going to help to screen for any sort of STI more quickly is gonna benefit gay community, I think. Any research to slow down syphilis I think any gay man will be happy to do whatever it takes. That’s my opinion.”* —Participant 1, living with HIV
Challenges of anal self-examination as a means for syphilis detection	*“So, I think if you could learn to do it yourself and check I think that would take a lot of worry out of having to think I have to go to the doctor to get that checked… but you also don’t want to go the other way where then people go I don’t need to go see a doctor for a screen because I’m doing it myself. So, it’s sort of a double edge sword in a way.”* —Participant 11, living with HIV
Challenges of anal self-examination as a means for syphilis detection	*“..I kind of think the gay community would totally be up for self-inspecting, but I don’t think the risk of STD contraction would go down, because the general … I think the general consensus is so many people are just having unprotected sex now, because of PrEP. So yes, I think … a lot of the gay community would feel comfortable doing it, but I don’t think it would stop or limit (STI risk) …”* —Participant 4, not living with HIV and taking PrEP

#### Acceptable to perform anal self-examination if recommended by a health professional

Men who had never performed or infrequently performed anal self-examination reported that they would be willing to start regularly examining their anus for signs of syphilis if it was recommended by a health professional.

Most men generally believed that anal self-examination for syphilis detection would be acceptable in the wider MSM community if recommended by a healthcare professional. Some men expressed that any tests or methods that could screen STI quickly would be beneficial to MSM community and MSM would be happy to do whatever it takes to reduce the risk of syphilis infection (See Table 6 for example quotes).

#### Open to perform anal self-examination when proven effective

A few men reported they would need more evidence on the effectiveness of anal self-examination as a screening tool for syphilis before performing a self-exam regularly.

*“…Unless they told me the reason to do it, like why am I doing this, I wouldn’t really do it.”*

—Participant 8, not living with HIV and taking PrEP

#### Already performing regular anal self-examination for health reasons

Whilst men who were already performing anal self-examination regularly found it acceptable to perform anal self-examination for detection of syphilis, a couple of these men raised concerns about being over reliant or confident in their own assessment. Specifically, these men were worried that they may detect an abnormality that they think is unimportant and therefore delay seeking healthcare when perhaps they should. For this reason, these men had a preference to have an examination by health professionals in the event of developing anal symptoms (see Table 6 for example quotes).

#### Preference for a health professional performing anal examination rather than self-examination

A number of men preferred a health professional to perform anal examination over anal self-examination including those who were prepared to perform anal self-examination if recommended by a health professional. Their concerns centred around a lack of knowledge and confidence in differentiating normal and abnormal examination, uncertainty not knowing what to look for during the examination and personal preference of having a trained professional performing the examination (see Table 6 for example quotes). Moreover, one participant highlighted having options, such as examination by a health professional, in addition to routine STI screening were important for men who were unable to perform anal self-examination.

*“There would probably be a group of gay men who you could sit down and have this conversation with for half an hour [about anal self-examination], who would then go away and just go, “I can’t deal with that.” So, they’re the types of people that would need to be engaging with their GP to do the test, rather than themselves… I think having those two key options is really, really important…”*

—Participant 6, living with HIV

#### Challenges of anal self-examination as a means for syphilis detection

Despite most men expressing a willingness to perform anal self-examination if recommended by health professionals, some men reported potential challenges that could be encountered in implementing anal self-examination for syphilis detection.

A few men suggested that some men who were top only would be apprehensive about performing anal self-examination although two men who mostly practised insertive or top anal sex in our study were open to perform anal self-examination if proven effective.

*“… it would be interesting to hear people that are tops, how often they would do it, if they’re top only… because I feel they would be more apprehensive about this versus either a bottom or a versatile person doing it.”*

—Participant 11, living with HIV

A few expressed concerns of placing a burden on health care services due to frequent presentations whenever MSM had concerning findings on anal self-examination for non-STI related anal problems. On the other hand, one participant counter-argued that men could become complacent with the false sense of security from regular self-examination thinking there were no problems in their anus, hence leading to missing anal pathologies or not performing regular STI screening as recommended (see Table 6 for examples). Although most men generally felt that gay men would be acceptable to performing anal self-examination, some men reported their view that anal self-examination is unlikely to reduce STI rates among gay men (see Table 6 for example quotes) suggesting the use of anal self-examination as an adjunct to existing measures to reduce STI rates and transmission.

## Discussion

This is the first qualitative study to investigate the attitudes and preferences of MSM regarding anal self-examination as a potential screening method to detect anal syphilis lesions. More than half of participants were already performing anal self-examination regularly or had performed anal self-examination at least once previously, largely for health reasons, with the most common concern being STI. A smaller number of men had performed anal self-examination for non-health-related reasons, primarily while pleasuring themselves. Taken together, the majority of men in our study were already familiar with performing anal self-examination and had some degree of practice, though most were not familiar with the terminology ‘anal self-examination’. Importantly, the majority of the participants reported they would be willing to perform anal self-examination regularly for the detection of syphilis if it was recommended by health professionals and/or proven effective. Some men, however, mostly those who infrequently performed or never performed anal self-examinations, were not confident with their ability to detect abnormalities during self-examination and would not be comfortable adopting the practice, suggesting further training and education is required if anal self-examination is to be recommended and taken up.

It is not known if regular anal self-examination will result in the detection of primary anal syphilis, and therefore studies evaluating the efficacy of anal self-examination for syphilis detection are required. Anal self-examination has been studied among HIV-positive MSM aged over 50 for anal cancer detection and was shown to be a cost-effective screening method in MSM living with HIV [17, 33, 34]. Our research shows men who practise receptive or versatile anal sex may be willing to adopt such a screening practice if it was recommended and came with education on what to look for during the examination, particularly signs of syphilis in the anus. This acceptability is consistent with findings from other studies, which show that self-STI testing and self-collection of anal swabs are highly acceptable among MSM [35, 36]. Moreover, there has not been any work in literature on anal self-examination as part of routine sexual health screening which suggests that some men might have preferred a health professional to perform anal examination over self-examination due to unfamiliar topic.

Findings suggest that a number of men were not familiar with the term “anal self-examination” even amongst those who were already performing the examination indicating it may be helpful for clinicians to use visual aids such as pictures or videos to clarify what anal self-examination is in future promotions or studies related to anal self-examination. Nonetheless, most men had had the experience of performing anal self-examination to some degree and it is likely that the concept of anal self-examination is not new to most MSM who practice receptive anal sex. This knowledge is likely to be useful in encouraging such practice in the future if anal self-examination is shown to be effective in detecting anal syphilis lesions.

While our study findings showed that some men would not be comfortable with a partner anal examination, some were open to partner examination, therefore, clinicians could consider advising MSM to have a partner help check their anus if comfortable in future. Additionally, some concerns were expressed about not seeking medical advice for abnormal findings on anal self-examination due to a misplaced sense of security. It is important that MSM have a good understanding of anal self-examination and signs and symptoms that might prompt them to seek medical advice before advising them of anal self-examination. Reinforcing their STI knowledge and encouraging ongoing regular testing will ensure that these men would remain engaged with sexual health services.

In this study, we anticipated that men living with HIV may be more familiar with the concept of anal self-examination due to the promotion of anal cancer screening in people living with HIV for aged 50 and above [18, 19, 37, 38]. However, not all men living with HIV might be aware of anal cancer screening using digital rectal examination administered either by health professionals or themselves. It would be important therefore, that any education of anal self-examination should be inclusive of MSM living with HIV infection as they are more likely to benefit from practising anal self-examination for a dual screening of anal cancer and syphilis.

Interestingly, most men indicated a preference to learn self-examination from health professionals or credible online resources from trusted health organisations implying the significant role of health professionals and health organisations in providing education around sexual health related matters. Multiple methods could be employed to educate MSM about anal self-examination, such as a sexual health doctor initiating a conversation about anal self-examination during consultations and introducing patients to credible online resources.

Our study investigated the views of MSM using a qualitative approach, which allowed for deeper exploration of the acceptability and willingness to perform anal self-examination as well as the identification of potential barriers and challenges of anal self-examination. There are a number of limitations to our study. First, many participants were already practising anal self-examination and likely to be more conscious of sexual health and STIs which may have presented a biased towards accepting anal self-examination for syphilis detection. However, these men were largely not practising anal self-examination for syphilis and their insights into adopting the practice were thus relevant, as was understanding their experiences around why and how they adopted this practice. It is possible, however, that a sample of MSM who rarely or never practice anal self-examination may have differed in their views and acceptance of the practice. Second, participants were recruited from a sexual health centre/clinic and thus their attitudes toward anal self-examination may reflect those of men who are more health conscious and thus are more willing to perform regular anal self-examination. Third, because our participants were recruited from a sexual health centre, they may be more inclined than members of the general population to prefer information about self-examination from health professionals. Fourth, we found men were not familiar with the term “anal self-examination” although most of them had performed similar practices before. However, we did not explore what their preferred or appropriate terminology would be for anal self-examination. This should be addressed in the future studies relating to anal self-examination. Finally, anal self-examination had its inherent limitation which is that rectal syphilis lesions are much harder to detect than anal lesions due to the limited physical accessibility and this was not addressed in our study as we were not evaluating the effectiveness of the anal self-examination. However, educating men about anal anatomy and explaining possible missed detection for rectal lesions in future studies or health promotions should encourage men to continue regular 3-monthly STI screening, including syphilis serology.

## Conclusions

Many men who practise receptive anal sex find it acceptable to perform anal self-examination and would be willing to adopt regular anal self-examination for detecting syphilis lesions if it was recommended by a healthcare professional. Further research is needed to evaluate the acceptability, adherence, and effectiveness of anal self-examination in MSM.

## Supplementary Information

**Additional file 1.** Qualitative research review guidelines - RATS

**Additional file 2.** Anal self-examination positions

## Data Availability

The datasets used and/or analysed during the current study are available from the corresponding author on reasonable request.

## Abbreviations

MSM: Men who have sex with men
HIV: Human immunodeficiency virus
PrEP: Pre-exposure prophylaxis for HIV infection
STI: Sexually transmitted infections
ASE: Anal self-examination
