# Supplementary material for: Exploring the attitudes of men who have sex with men on anal self-examination for early detection of primary anorectal syphilis: a qualitative study
Source: BMC Infect Dis. 2021 Sep 20;21:982. doi: 10.1186/s12879-021-06686-4 (PMC8453991; doi:10.1186/s12879-021-06686-4)
Supplement: Supplementary file 1 — Additional file 1. Qualitative research review guidelines - RATS [file 12879_2021_6686_MOESM1_ESM.pdf]

Table S1. Qualitative Research Review Guidelines (RATS) checklist.

Qualitative Research Review Guidelines – RATS [1]		
**Ask this of the manuscript**	**This should be included in the manuscript**	**Where item has been addressed**
**R – Relevance of study design**		
Is research question interesting?	Research question explicitly stated	Page 3&4<br>1 and objectives provided on pages 2 and 6
Is research question relevant to clinical practice, public health, or policy?	Research question justified and linked to the existing knowledge base (empirical research, theory, policy)	Page 3&4, relevant to clinical practice, guideline/policy and public health
**A – Appropriateness of qualitative method**		
Is qualitative methodology the best approach for the study aims?<br><br>*Interviews*: experience, perceptions, behavior, practice, process	Study design described and justified e.g., why was a particular method (i.e., interviews) chosen?	Pages 3<br><br>Interviews: perceptions, practice, attitudes
**T – transparency of procedures**		
Sampling<br>Are the participants selected the most appropriate to provide access to the type of knowledge sought by the study?<br><br>Is the sampling strategy appropriate?	Criteria for selecting the study sample justified and explained<br>*theoretical*: based on pre conceived or emergent theory<br>*purposive*: diversity of opinion<br>*volunteer*: feasibility, hard-to-reach groups	Page 4 to 6
Recruitment<br>Was recruitment conducted using appropriate methods?<br><br>Is the sampling strategy appropriate?<br><br>Could there be selection bias?	Details of how recruitment was conducted and by whom<br><br>Details of who chose not to participate and why	Pages 4 through to 6<br><br>Pages 4 through to 6 (exclusion criteria page 5)<br><br>Possible, addressed in discussion (limitations of study, page 21) and page 6

Data collection<br>Was collection of data systematic and comprehensive?	Method (s) outlined and examples given (e.g., interview questions)	Page 5 to 6, Supplementary material
Are characteristics of study group and setting clear?	Study group and setting dearly described	Page 5 to 6
Why and when was data collection stopped, and is this reasonable?	End of data collection justified and described	Page 5 to 6
Role of researchers<br>Is the researcher(s) appropriate?	Do the researchers occupy dual roles (clinician and researcher)?	Page 6 (yes)
How might they bias (good and bad) the conduct of the study and results?	Are the ethics of this discussed?<br>Do the researcher(s) critically examine their own influence on the formulation of the research question, data collection, and interpretation?	Page 6 (reflection addressed)
Ethics<br>Was informed consent sought and granted?	Informed consent process explicitly and dearly detailed	Page 4 to 6
Were participants'' anonymity and confidentiality ensured?	Anonymity and confidentiality discussed	Page 6-7
Was approval from an appropriate ethics committee received?	Ethics approval cited	Page 4
**S - Soundness of interpretive approach**		
Analysis<br>Is the type of analysis appropriate for the type of study?	Analytic approach described in depth and justified	Pages 7-8
Thematic: exploratory, descriptive, hypothesis generating<br>*Framework:* e.g. policy<br>*Constant comparison/grounded theory:* theory generating, analytical	Indicators of quality:<br>Description of how themes were developed from the data (inductive or deductive)<br>Evidence of alternative explanations being sought<br>Analysis and presentation of negative or deviant cases	Pages 7-8
Are the interpretations dearly presented and adequately supported by the evidence?	Description of the basis on which quotes were chosen<br>Semi-quantification when appropriate	Pages 7-8, Tables 3 to 6, REF 30-33
Are quotes used and are these appropriate and effective?	Illumination of context and/or meaning, richly detailed	Pages 8 to 16, Tables 3 to 6
