# Supplementary material for: Exploring the attitudes of men who have sex with men on anal self-examination for early detection of primary anorectal syphilis: a qualitative study
Source: BMC Infect Dis. 2021 Sep 20;21:982. doi: 10.1186/s12879-021-06686-4 (PMC8453991; doi:10.1186/s12879-021-06686-4)
Supplement: Supplementary file 2 — Additional file 2. Anal self-examination positions [file 12879_2021_6686_MOESM2_ESM.pdf]

Supplementary Table 1

Positions of anal self-examination and preferences

Positions of ASE	Number of participants who preferred this position	Main preferred location
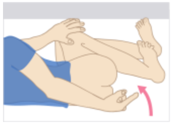	11	Bedroom
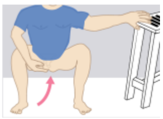	7	Shower/toilet
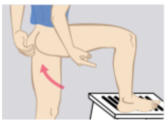	7	Shower/toilet
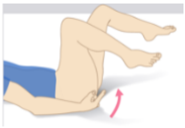	2	Bedroom

NB: Some participants preferred or use multiple positions; therefore, the total would not add up to 20.
